# Ndfip2 is a potential regulator of the iron transporter DMT1 in the liver

**DOI:** 10.1038/srep24045

**Published:** 2016-04-06

**Authors:** Natalie J. Foot, Kelly M. Gembus, Kimberly Mackenzie, Sharad Kumar

**Affiliations:** 1 Centre for Cancer Biology, University of South Australia, c/o SA Pathology, PO Box 14, Rundle Mall, Adelaide, SA 5001, Australia

## Abstract

The regulation of divalent metal ion transporter DMT1, the primary non-heme iron importer in mammals, is critical for maintaining iron homeostasis. Previously we identified ubiquitin-dependent regulation of DMT1 involving the Nedd4 family of ubiquitin ligases and the Ndfip1 and Ndfip2 adaptors. We also established the *in vivo* function of Ndfip1 in the regulation of DMT1 in the duodenum of mice. Here we have studied the function of Ndfip2 using Ndfip2-deficient mice. The DMT1 protein levels in the duodenum were comparable in wild type and *Ndfip2*^*−/−*^ mice, as was the transport activity of isolated enterocytes. A complete blood examination showed no significant differences between wild type and *Ndfip2*^*−/−*^ mice in any of the hematological parameters measured. However, when fed a low iron diet, female *Ndfip2*^*−/−*^ mice showed a decrease in liver iron content, although they maintained normal serum iron levels and transferrin saturation, compared to wild type female mice that showed a reduction in serum iron and transferrin saturation. *Ndfip2*^*−/−*^ female mice also showed an increase in DMT1 expression in the liver, with no change in male mice. We suggest that Ndfip2 controls DMT1 in the liver with female mice showing a greater response to altered dietary iron than the male mice.

Iron is an essential element for the normal functioning of cells and for animal survival. Iron uptake and metabolism are complex and highly regulated processes, which involves a number of enzymes, transporters and regulatory proteins^1^. Iron levels are tightly controlled, as too little iron results in anemia, whereas elevated iron levels can lead to tissue damage and fibrosis due to the generation of reactive oxygen species. The divalent metal-ion transporter-1 (DMT1; also known as DCT1 and Slc11a2) is the primary importer of non-heme iron, and is ubiquitously expressed throughout the body, with highest expression in the proximal duodenum (main site of iron uptake)^2^ and liver (main site of iron storage)^3^. The importance of DMT1 in the iron regulatory cycle is demonstrated by the fact that mutations in both humans and rodents result in severe hypochromic microcytic anemia^4^,^5^,^6^,^7^.

DMT1 expression and function are regulated by dietary iron availability through transcriptional and post-translational mechanisms, including modification by ubiquitination^8^. Ubiquitination is well known for its role in regulating ion channels and transporters^9^. Ubiquitin ligases, which catalyse the transfer of ubiquitin to the substrate, confer specificity to the system by targeting specific proteins. The Nedd4 family are members of the HECT class of ubiquitin ligases, which bind to their targets directly through WW domains on the ligase interacting with proline-rich PPxY (PY) motifs on the substrate^10^. Members of this family, particularly Nedd4 and Nedd4-2, are involved in regulation of membrane proteins, including ion channels and transporters^10^. A well characterised example of this is the regulation of the epithelial sodium channel (ENaC) by Nedd4-2, where the WW domains of the ligase directly interacts with the PY motifs present within the three ENaC subunits^11^,^12^,^13^. In some cases the substrates of the Nedd4 family lack PY motifs and the interaction can be mediated through adaptor or accessory proteins. Two such adaptor proteins are Ndfip1 and Ndfip2, initially identified by us as Nedd4 WW domain interacting proteins^14^,^15^. In previous studies, we found that DMT1 is ubiquitinated by the Nedd4 family members Nedd4-2 and WWP2 *in vitro*, and this requires Ndfip1 and/or Ndfip2^8^,^16^,^17^. In mice deficient in Ndfip1 we found increased duodenal DMT1 levels and activity, leading to increased iron uptake and storage in liver^16^,^18^. Interestingly, Ndfip1 is also known to be upregulated in neurons in response to divalent metals, such as cobalt, and protects neurons against metal ion toxicity via Nedd4-2-mediated DMT1 ubiquitination and down regulation^17^. Furthermore, Ndfip1 has been shown to regulate DMT1-dependent iron levels in the brain and play a role in Parkinson’s disease pathology^19^. These studies indicate that Ndfip1 plays a critical role in iron homeostasis *in vivo*. However, the *in vivo* function of Ndfip2 remains unknown.

In this study we report that in contrast to Ndfip1, Ndfip2 is not required for duodenal DMT1 regulation but is likely to play a role in the regulation of DMT1 in the liver, particularly in female mice, which appear to have an increased sensitivity for iron perturbations. We suggest that both Ndfip1 and Ndfip2 are required for regulating DMT1 *in vivo*, but they function in a context and tissue specific manner.

## Results

### Characterisation of *Ndfip2* ^−/−^ mice

**Figure 1:**
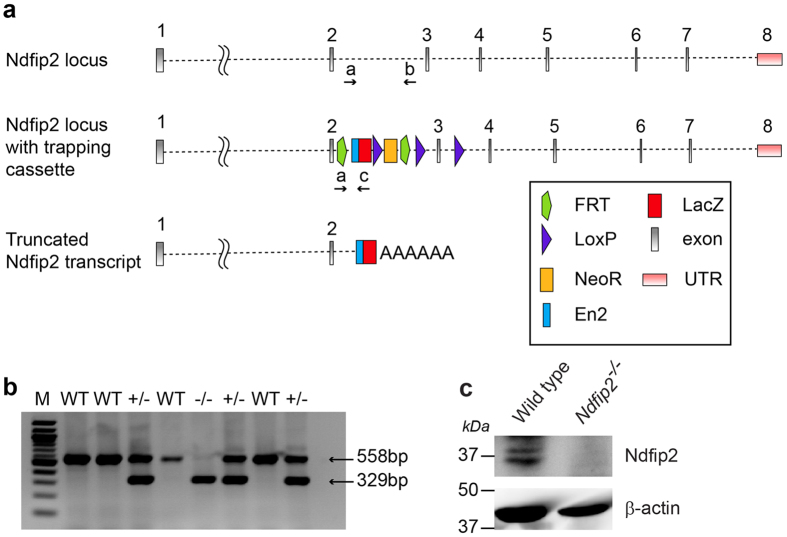
The generation of mice homozygous for a disrupted *Ndfip2* gene. (**a**) The knockout mice were generated by EUCOMM. Location of the inserted trapping cassette within intron 2 of the *Ndfip2* gene, resulting in a truncated transcript that encodes exons 1 and 2. The common forward primer (**a**), wild type reverse primer (**b**) and knockout reverse primer (**c**) are indicated. (**b)** PCR results from one litter produced by two heterozygous parents showing the sizes of the wild type (558 bp) and knockout (329 bp) bands. WT, wild type; M, marker. (**c)** Immunoblot showing the absence of Ndfip2 protein in *Ndfip2*^*−/−*^ spleen tissue.

The *Ndfip2*^*−/−*^ mice used in this study were generated by The European Conditional Mouse Mutagenesis Program (EUCOMM) by inserting a targeting vector cassette into intron 2 of the *Ndfip2* gene (Fig. 1a). This disruption results in a truncated transcript expressing exon 1 and 2 fused to *lacZ* (Fig. 1a). To genotype these mice, PCR was performed using a triplex reaction containing the primers listed in Supplementary Table 1. PCR with primers “a” and “b” produces a 558 bp band, indicating the presence of the Wild type (WT) locus. PCR with primers “a” and “c” yielded a band of 329 bp, indicating the disrupted locus (Fig. 1b). Immunoblotting confirmed that no detectable Ndfip2 protein was produced in the *Ndfip2*^*−/−*^ samples (Fig. 1c), showing that the knockout results in a null allele.

*Ndfip2*^*−/−*^ mice have no developmental defects or gross anatomical abnormalities, and they live to an age comparable to WT mice (*Ndfip2*^*−/−*^ mice were followed up to 24 months). This is in contrast to *Ndfip1*^*−/−*^ mice, which develop a severe inflammatory phenotype and do not survive past 14 weeks of age^20^.

### Ndfip2 is not required for DMT1 regulation in the duodenum

**Figure 2:**
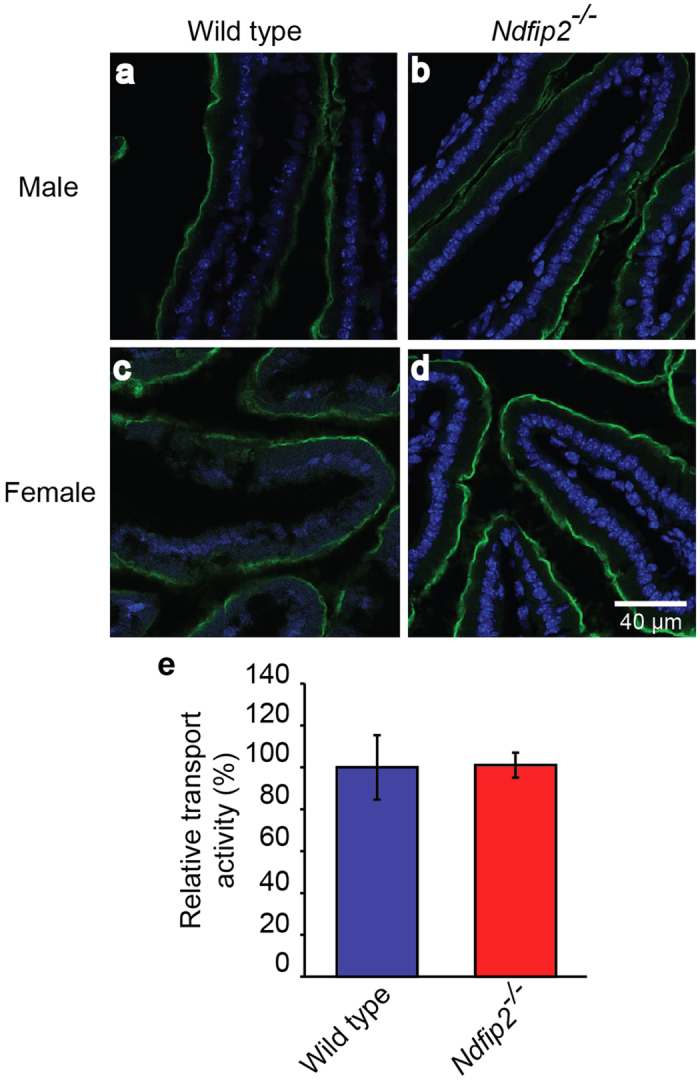
*Ndfip2*-deficient mice show normal DMT1 expression and activity. DMT1 expression on the apical surface of the duodenum (green) of (**a**) male wild type and (**b**) *Ndfip2*^*−/−*^ mice, and (**c**) female wild type and (**d**) *Ndfip2*^*−/−*^ mice fed a low iron diet for three weeks. Representative images from n = 4. Blue shows DAPI staining of the nuclei. (**e**) DMT1 relative transport activity as measured by the fluorescence quenching assay in enterocytes isolated from wild type and *Ndfip2*^*−/−*^ mice fed a low iron diet show no significant difference. Data represented as mean ± s.e.m.; n = 5.

*In vitro* studies using CHO cells stably expressing Myc-tagged DMT1 have shown that overexpression of both Ndfip1 and Ndfip2 results in lower DMT1 activity^16^. Additional studies showed that *Ndfip1*^*−/−*^ mice have increased iron deposits in their livers^16^ and that Ndfip1 is important for DMT1 regulation in the duodenum under low iron conditions^18^. However, the *in vivo* role for Ndfip2 has not been investigated. To determine the importance of Ndfip2 in DMT1 regulation in mice, we fed *Ndfip2*^*−/−*^ mice either a normal or low iron diet for three weeks. Immunostaining of duodenum of male mice fed a low iron diet showed no difference in DMT1 levels between WT and *Ndfip2*^*−/−*^ mice (Fig. 2a,b). To determine whether the effect of Ndfip2 on DMT1 regulation in the duodenum was sex-specific, we also stained duodenum from female mice; these also showed no significant differences between WT and knockout mice (Fig. 2c,d). Furthermore, isolated enterocytes did not show any increase in transport activity in *Ndfip2*^*−/−*^ mice (Fig. 2e). This is in contrast to the *Ndfip1*^*−/−*^ mice, which show an increase in DMT1 staining in the duodenum and an increase in transport activity in isolated enterocytes^18^. Taken together, these data suggest that Ndfip1 and Ndfip2 function differently in regulating DMT1 *in vivo*.

As perturbations in iron homeostasis often manifest in abnormal hematological parameters, we performed a complete blood examination on WT and *Ndfip2*^*−/−*^ mice. Again, unlike in the *Ndfip1*^*−/−*^ mice^18^, none of the parameters showed a significant difference between the WT and *Ndfip2*^*−/−*^ mice in either males or females (Table 1). Interestingly, male mice seemed less affected by the low iron diet than females. While there was a significant decrease in mean corpuscular volume (MCV), mean cell hemoglobin (MCH) and red cell distribution width (RDW) with a corresponding increase in red blood cell (RBC) count in mice on the low iron diet as would be expected in iron-deficient anemia, there was no significant difference in hemoglobin or hematocrit as there is in the females, indicating that male mice are perhaps less sensitive to perturbations in iron levels than females.

### Ndfip2 maintains iron homeostasis in female mice by regulating DMT1 in the liver

**Figure 3:**
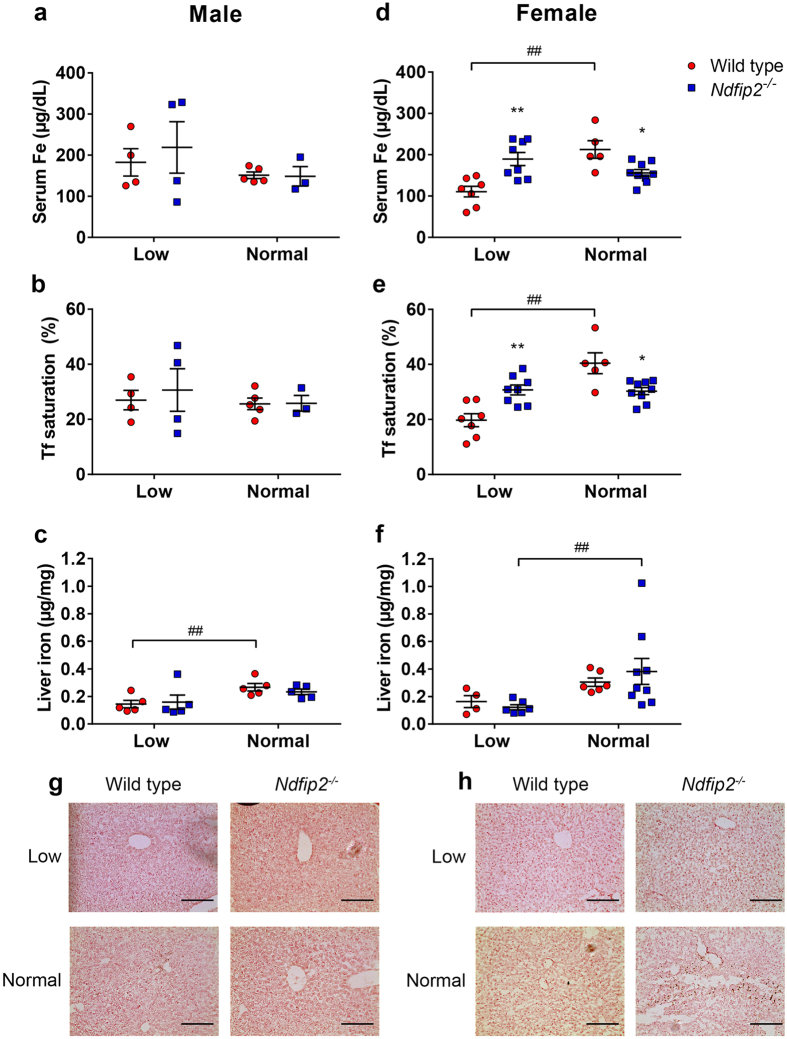
Sex-specific changes in serum iron, transferrin saturation and liver iron measurements in mice fed a low or normal iron diet. (**a**,**d**) Serum iron levels and (**b**,**e**) transferrin (Tf) saturation in male and female wild type and *Ndfip2*^*−/−*^ mice fed a normal or low iron diet. Male mice show no significant differences in serum iron or Tf saturation between normal and low iron diet in either wild type or knockout mice. Wild type female mice show a significant decrease in serum iron (^##^p < 0.0001) and Tf saturation (^##^p < 0.0001) when fed a low iron diet, but *Ndfip2*^*−/−*^females are able to maintain their levels when challenged. *represents a significant difference in serum iron (p = 0.0232) and Tf saturation (p = 0.008) between wild type and knockout females fed a normal diet. **represents a significant difference in serum iron (p = 0.0008) and Tf saturation (p = 0.0021) between wild type and knockout females fed a low iron diet. (**c**,**f**) Liver iron levels in wild type and *Ndfip2*^*−/−*^mice fed a normal or low iron diet. Wild type males show a significant reduction in liver iron levels when fed a low iron diet (^##^p = 0.0425), but *Ndfip2*^*−/−*^males are able to maintain liver iron levels. *Ndfip2*^*−/−*^ females show a decrease in liver iron levels (^##^p = 0.0276). Red circles, wild type; blue squares, *Ndfip2*^*−/−*^. Data represent mean ± s.e.m.; n = 4–8. (**g**) Liver iron levels in male and (**h**) female mice as measured by Perl’s Prussian blue staining. *Ndfip2*^*−/−*^ female mice fed a normal diet show significant iron deposits compared to wild type mice, and male mice do not show any significant staining.

To assess the effect of Ndfip2 on iron transport and storage, we measured serum iron and transferrin saturation, as well as iron levels in the liver. In male mice, there were no significant differences between the normal and low iron diet in serum iron or transferrin saturation in either the WT or *Ndfip2*^*−/−*^ mice (Fig. 3a,b). Liver iron levels were significantly decreased in male WT mice when fed a low iron diet, but *Ndfip2*^*−/−*^ mice show no such effect (Fig. 3c). Female WT mice showed a significant decrease in serum iron and transferrin saturation when fed a low iron diet, while the *Ndfip2*^*−/−*^ mice showed no significant decrease (Fig. 3d,e). Further to this, when fed a low iron diet, female WT mice showed no change in liver iron levels, but liver iron levels increased in female *Ndfip2*^*−/−*^mice on a normal diet (Fig. 3f). Differences in liver iron levels were confirmed by Perl’s staining in liver sections, which demonstrated that male mice have very little iron deposition in the liver regardless of dietary iron levels (Fig. 3g), whereas female *Ndfip2*^*−/−*^mice on a normal diet show increased liver iron staining compared to WT mice (Fig. 3h).

**Figure 4:**
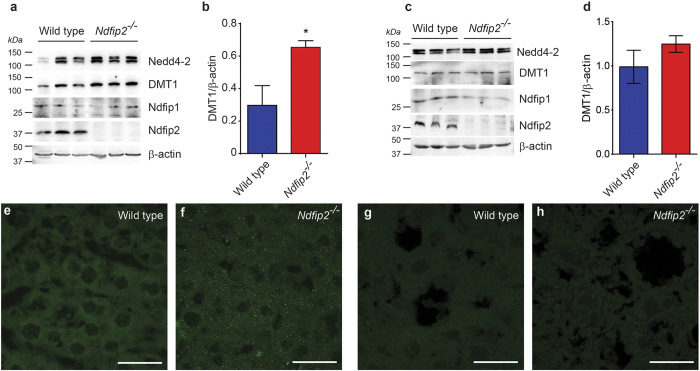
DMT1 expression is increased in the liver of female *Ndfip2*^*−/−*^mice, and there is no compensation for loss of Ndfip2 by Ndfip1 or Nedd4-2. (**a**) Western blot showing levels of Ndfip1, Nedd4-2, DMT1 and Ndfip2 in female and (**c**) male liver protein lysates. There is no change seen in the levels of Ndfip1 or Nedd4-2 in *Ndfip2*^*−/−*^ samples, indicating no compensation is occurring. DMT1 levels are increased in *Ndfip2*^*−/−*^ female mice, but unchanged in male mice, suggesting that Ndfip2 is important for liver DMT1 regulation in females. β-actin acts as a loading control. (**b**,**d**) Quantitation of DMT1 expression relative to β-actin in liver samples from (**a**,**c**) showing significant upregualation of DMT1 in female *Ndfip2*^*−/−*^ mice. Data expressed as mean ± s.e.m. (n = 3), *p = 0.04. (**e**,**f**) DMT1 expression in liver sections from wild type and *Ndfip2*^*−/−*^female mice showing an increase in DMT1-positive puncta in the *Ndfip2*^*−/−*^liver. (**g**,**h**) DMT1 expression in liver sections from male mice showing no significant difference between wild type and *Ndfip2*^*−/−*^ mice. Representative images of n = 4.

Since Ndfip2 did not appear to be required for DMT1 regulation in the duodenum, but seemed to modulate liver iron levels, we next assessed DMT1 regulation in the liver. Total DMT1 levels were increased in liver lysates of female *Ndfip2*^*−/−*^mice (Fig. 4a, quantitation in Fig. 4b) but not in male mice (Fig. 4c, quantitation in Fig. 4d), and liver sections from female *Ndfip2*^*−/−*^mice showed an increase in DMT1-labelled puncta compared to WT (Fig. 4e,f), with no change in male mice (Fig. 4g,h), suggesting that Ndfip2 is important for DMT1 regulation in the liver in female mice.

Ndfip1 and Ndfip2 share 52% sequence identity and 79% similarity^15^ and may function redundantly. It is also possible that Ndfip1 may be upregulated to compensate for the loss of Ndfip2. We therefore assessed the levels of Ndfip1 protein in liver lysates from WT and *Ndfip2*^*−/−*^ male and female mice, and found that Ndfip1 levels were unaltered in the *Ndfip2*^*−/−*^ mice (Fig. 4a,c). As Ndfips are known to recruit the Nedd4 family of ubiquitin ligases to regulate DMT1^8^,^16^,^17^, we also checked the levels of Nedd4-2 but found no significant change (Fig. 4a,c).

**Figure 5:**
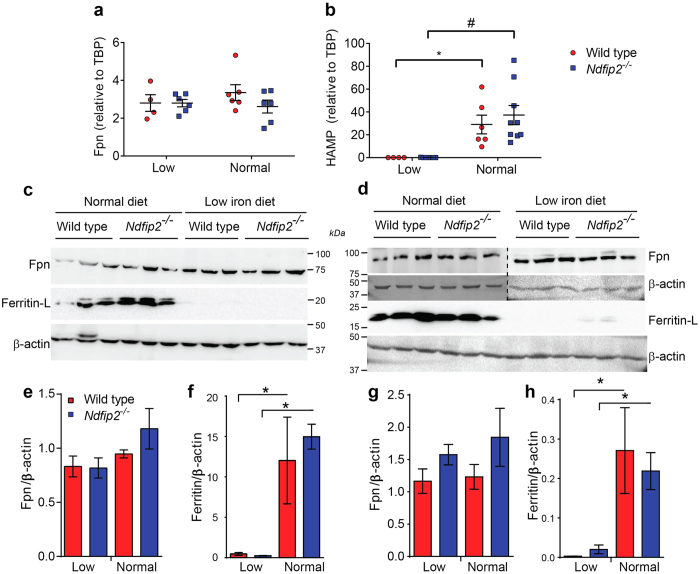
Ferroportin, ferritin and hepcidin levels are unaffected by loss of Ndfip2. (**a**) Ferroportin (Fpn) and (**b**) hepcidin (HAMP) mRNA levels expressed relative to TATA Box Binding Protein (TBP) are not significant between wild type and *Ndfip2*^*−/−*^ mice fed either a low iron or normal diet. Hepcidin levels significantly decrease as expected in both the wild type and *Ndfip2*^*−/−*^ mice fed a low iron diet (*p = 0.0402, ^#^p = 0.0009). (**c**) Fpn and ferritin (light chain) protein levels in liver lysates from wild type and *Ndfip2*^*−/−*^ female and (**d**) male mice showing no difference in expression between genotypes. The dotted line in **d** indicates that the right and left sides of the blots are from different gels. This is quantitated in (**e**–**h**) showing no significant difference between genotypes. *indicates significant differences in ferritin levels between diets as expected (p < 0.05). Red circles, wild type; blue squares, *Ndfip2*^*−/−*^. Data expressed as mean ± s.e.m, n = 4–9.

Ferroportin (Fpn) is the primary iron exporter in cells and is responsible for iron export from both enterocytes and hepatocytes^2^. Hepcidin is a small peptide produced by the liver in response to altered iron levels^21^, and ferritin complexes store excess iron molecules to inhibit their toxic effects and for release when required^22^. To determine whether Fpn, hepcidin or ferritin expression was altered in these mice, we measured mRNA and protein levels in livers from mice fed either a low iron or normal diet. While hepcidin responded as expected by decreasing under low iron conditions, neither Fpn nor hepcidin mRNA levels were significantly different between WT and *Ndfip2*^*−/−*^mice on either diet (Fig. 5a,b). Ferritin also responded as expected by decreasing in mice on a low iron diet, but both ferroportin and ferritin protein levels were unchanged between genotypes (Fig. 5c,d, quantitated in Fig. 5e–h).

## Discussion

Our previous work has demonstrated that Ndfip1 is an important regulator of DMT1 in mice^16^,^18^. To determine whether Ndfip2 is also necessary for DMT1 regulation, we performed the low iron feeding study using *Ndfip2*^*−/−*^ mice. Unlike the *Ndfip1*^*−/−*^ mice, *Ndfip2*^*−/−*^ mice showed no significant difference in duodenal DMT1 expression or any of the hematological parameters measured; however, hepatic DMT1 levels were increased. In previous studies both Ndfip1 and Ndfip2 were found to interact with and ubiquitinate DMT1 in a heterologous *in vitro* system^16^, and Ndfip1 was found to be important for DMT1 regulation primarily in the duodenum, but also in the liver^18^. The results reported here suggest that Ndfip2 is dispensable for duodenal DMT1 regulation, but it may be required for liver DMT1 regulation. Both Ndfip1 and Ndfip2 are expressed in the liver (Fig. 4), but the predominance of Ndfip2 expression over Ndfip1 may, at least in part, explain why Ndfip2 appears to be the main regulator in the liver. DMT1 has four major isoforms, differing at both the 5’ and 3’ termini (designated 1A/B and ±IRE, respectively)^8^. These isoforms are differentially expressed in various tissues, with the 1A/+IRE isoform being predominant in the duodenum^8^, and the 1B isoforms being the major ones in the liver^3^,^23^. It is possible that Ndfip1 and Ndfip2 regulate alternative isoforms, which gives rise to their apparent tissue-specific functions. Further studies would be required to test this hypothesis.

Interestingly, on low iron diet *Ndfip2*^*−/−*^ female mice were able to maintain steady serum iron levels and transferrin saturation, whereas WT mice showed a reduction in these parameters; this was coupled with an increase in hepatic DMT1 expression in the *Ndfip2*^*−/−*^ mice. Hepatic DMT1 is responsible for transporting iron from the endosome into the intracellular space, where it is then able to be either released into circulation or stored bound to ferritin^24^. The higher levels of DMT1 in the liver resulting from the loss of Ndfip2 actually place these mice at a competitive advantage over WT mice in that there is more available iron to be released (as indicated by the reduction in liver iron levels) to compensate for the reduction in iron taken up from the diet. The stronger phenotype in the females may reflect a possible increase in sensitivity to iron levels suggested by hematological data, both in our study (Table 1), and in work previously reported^25^. Earlier studies have also reported sex-specific changes in iron parameters in mice on both iron-deficient and iron overload diets^26^,^27^. The age of the mice used in this study may also account for the sex-dependent differences we observe, as changes in hematological parameters and tissue iron levels are potentially sex-dependent in young mice^28^,^29^. Nevertheless, our data suggest that Ndfip2 is playing a role in DMT1 regulation in the liver, which is highlighted in female mice possibly due to an inherent sensitivity to iron perturbation.

Male *Ndfip2*^*−/−*^mice seem to be able to maintain liver iron stores, unlike the WT males that show a decrease in liver iron. This may be due to alterations in iron recycling by macrophages, which has been shown to be affected in cells and mice lacking the DMT1 homologue Nramp1^30^,^31^. It has also been suggested that both DMT1 and Nramp1 are necessary for the efficient recycling of iron by macrophages^32^. Therefore, if Ndfip2 is also a regulator of DMT1 in macrophages (including Kupffer cells in the liver), changes in iron recycling in *Ndfip2*^*−/−*^ mice may explain the differences in liver iron stores. Indeed the increased iron deposition in the female *Ndfip2*^*−/−*^ mice liver appears to be primarily in the Kupffer cells surrounding central veins (Fig. 3h). Further experiments to evaluate the effect of Ndfips on DMT1 and/or Nramp1 in macrophages and iron recycling would be a worthwhile avenue of future study.

In summary, this study suggests that Ndfip2 is involved in the regulation of DMT1 *in vivo*, and that while Ndfip1 and Ndfip2 function by the same mechanism of recruiting Nedd4 family ligases to ubiquitinate and degrade DMT1, due to their tissue-specific function, loss of Ndfip2 alleviates the phenotype associated with loss of dietary iron. This could have important consequences for the diagnosis and treatment of iron-deficiency anemia and other diseases associated with decrease iron uptake.

## Materials and Methods

### Animals

The *Ndfip2* knockout mice were purchased from the European Mouse Mutant Archive (EMMA). These mice were generated in a C57B6NTac background. The line originated from EUCOMM embryonic stem cell clone HEPD0550_6_G09 (International strain name B6NTac;B6N-Ndfip2^tm1a(EUCOMM)Hmgu^). This targeted mutation was created by the insertion of the L1L2_Bact_P cassette into position 105291701 of chromosome 14, which corresponds with intron 2 of the *Ndfip2* gene. The L1L2-Bact_P cassette in composed of an artificial exon (En2), a splice acceptor site, and the *lacZ* and *NeoR* genes, flanked by FRT and LoxP sites. Without the presence of Flp or Cre recombinases, this insertion results in a ubiquitous knockout (*Ndfip2*^*−/−*^) mouse. Animals were rederived, bred and maintained in SA Pathology Animal Care Facility and the University of South Australia Reid Animal Facility.

For iron feeding experiments, animals were fed *ad libitum* on a standard (164 mg/kg iron) or low iron (15 mg/kg) rodent diet (Specialty Feeds, Australia) for 3 weeks immediately following weaning. The studies were performed on 6-week-old littermates. All animal studies were approved by the institutional animal ethics and biosafety committees at SA Pathology and the University of South Australia, and were carried out according to the National Health and Medical Research Council guidelines.

### Genotyping

DNA was extracted from tail or ear fragments and PCR was performed using the KAPA Mouse HotStart Genotyping Kit (Geneworks, Australia), according to manufacturer’s instructions. To genotype *Ndfip2* mice, a common primer situated at the beginning of intron 2 was used for PCR with one reverse primer against the end of intron 2 and another found within the *lacZ* gene of the trapping cassette (Fig. 1a). Primer sequences are listed in Supplementary Table 1. This triplex reaction results in a WT band of 558 bp and a knockout band of 329 bp (Fig. 1b). The absence of Ndfip2 protein in the knockout mice was confirmed by immunoblotting (Fig. 1c).

### Antibodies and reagents

Rabbit anti-DMT1 designed against the fourth extracellular loop of mouse DMT1 was a generous gift from Dr Michael Garrick (University at Buffalo, New York)^33^. The production and affinity purification of a rabbit polyclonal against recombinant Ndfip1, Nedd4-2 and Ndfip2 have been described previously^14^,^15^,^34^. Commercial antibodies and reagents were purchased from the following suppliers: Donkey anti-rabbit AlexaFluor-488 and calcein-AM (Life Technologies), rabbit polyclonals anti-ferritin (light chain) and anti-ferroportin (Abcam), mouse monoclonal anti-β-actin (clone AC-15; Sigma-Aldrich), donkey anti-rabbit HRP and ECL™ Plex goat anti-mouse Cy5 (GE Healthcare). ECL prime (GE Healthcare) was used as the detection reagent for immunoblotting.

### Blood and serum analyses

Blood was collected via cardiac puncture and complete blood count was performed by the Department of Clinical Pathology, SA Pathology. Serum was separated from whole blood using a 0.8 ml Z serum Sep MiniCollect tube (Greiner bio-one, Austria) with centrifugation at 5000 rpm for 10 min. Serum iron and transferrin saturation were measured using a Randox Fe/UIBC kit according to manufacturer’s instructions.

### Immunohistochemistry and confocal microscopy

1–2 cm of proximal duodenum was fixed in Histochoice^TM^ (ProSciTech) before being cryoprotected in 30% sucrose in PBS and then embedded in Tissue-Tek OCT compound. 10 μm frozen sections were mounted on polysine slides, fixed with ice cold acetone for 10 min, air dried, then rehydrated with PBS. Sections were then blocked with 5% skim milk in PBS for 2 h at room temperature and then stained with rabbit anti-DMT1 overnight at 4 °C (1:500 in 5% skim milk in PBS). After washing in PBS, sections were incubated with rabbit AlexaFluor-488 (1:500) for 2 h, washed, counterstained with DAPI and mounted in ProLong Gold antifade (Invitrogen). Confocal images were captured using a Zeiss LSM 700 confocal microscope using a 40×/1.30 water differential interference contrast (DIC) M27 objective and digitized at a depth of 8 bits. The fluorescence was sequentially acquired for multiple channels to avoid emission spectral bleed-through.

### Immunoblotting

Proteins transferred to polyvinylidene difluoride membrane were immunoblotted with primary antibody diluted in TBST (137 mM NaCl, 2.7 mM KCl, 19 mM Tris, 0.1% Tween-20) overnight at 4 °C, followed by incubation with a horseradish peroxidase (HRP) or Cy5-conjugated secondary antibody. Visualization of Cy5 signal was carried out on a Typhoon FLA biomolecular imager (GE Healthcare) and HRP signals were detected on an ImageQuant LAS 4000 (GE Healthcare). Blots were quantitated using ImageJ software^35^.

### Enterocyte isolation and fluorescence quenching assay

Enterocytes from WT and *Ndfip2*^*−/−*^ mice were isolated as previously described^18^. Briefly, the proximal duodenum was removed and flushed through then washed with solution A (1.5 mM KCl, 96 mM NaCl, 27 mM sodium citrate, 8 mM KH_2_PO_4_, 5.6 mM Na_2_HPO_4_). The duodenum was minced in an enzyme cocktail (333 U/mL collagenase, 2.5 U/mL elastase, 10 μg/mL DNAse) in Krebs Ringer solution (120 mM NaCl, 24 mM NaHCO_3_, 5 mM HEPES [4-(2-hydroxyethyl)-1-piperazineethanesulfonic acid], 4.8 mM KCl, 1.2 mM MgSO_4_, 1.2 mM KH_2_PO_4_, 20 mM glucose, 1 mM CaCl_2_) and incubated while shaking at 37 °C for 30 min. Cells were filtered through a 40 μm filter and washed twice in Dulbecco modified Eagle medium (DMEM) supplemented with 10% fetal calf serum, 1% nonessential amino acids, 50 μM ß-mercaptoethanol, and 100 μM L-asparagine. To determine the relative transport activity of freshly isolated enterocytes, we used a fluorescence quenching assay as previously described^16^. Cells were loaded with 0.25 μM calcein-AM (Invitrogen) for 20 min at 37 °C in loading medium (DMEM supplemented with 20 mM HEPES). Cells were washed and resuspended in transport buffer (150 mM NaCl, 20 mM 2-(N-morpholino)ethanesulfonic acid) and fluorescence was recorded using a BMG FLUOStar Optima microplate reader (excitation 485 nm, emission 520 nm). Readings were taken every 2 seconds for the first 20 seconds to achieve a baseline reading, then every 0.5 second after injection of CoCl_2_ (final concentration, 100 μM; pH 6.7) for 150 seconds. Transport activity was calculated as the rate of fluorescence quenching (slope) observed in a 1-min period after the injection of metal ions and is displayed relative to wild-type enterocytes.

### Perl’s Prussian Blue staining with DAB intensification

Iron deposits in liver sections were visualised using the Perl’s Prussian Blue technique. Sections were rinsed in TBST, then incubated in methanol for 20 min, followed by incubation in Perl’s working solution (1% HCl, 2% potassium ferrocyanide) for 1 h. Staining was intensified using DAB peroxidase substrate kit with nickel chloride (Vector Laboratories, CA) following manufacturer’s instructions. Neutral red was used as a counterstain.

### RNA isolation and Quantitative PCR (qPCR)

RNA was extracted from liver tissue using TRIzol reagent (Life Technologies), and was reverse-transcribed with a High Capacity cDNA reverse transcription kit (Applied Biosciences). qPCR was performed on a Rotor-Gene™ 3000 (Qiagen) using RT2 Real-Time SYBR^®^ Green/ROX PCR Master Mix (Qiagen) as per the manufacturer’s instructions, using the following themocycler conditions: 95 °C for 10 min, followed by 40 cycles of 95 °C for 15 s, 60 °C for 30 s, 72 °C for 30 s with the primers listed in Supplementary Table 1. Expression was normalized to TBP by the 2^−ΔΔCt^ method using Rotor-Gene™ 6000 Software (v1.7, Qiagen) and Microsoft Excel software.

### Tissue iron quantitation

Small pieces of liver tissue were dried for 24 h at 85 °C after which they were weighed and digested in 25% nitric acid in a glass tube at 65 °C for 16 h. The samples were then diluted to 1% nitric acid and analysed by inductively coupled plasma mass spectroscopy (ICP-MS) by the Australian Water Quality Centre at SA Water. Values were normalized to dry weight.

### Statistics

Statistical analyses were performed using unpaired two-tailed t-tests or two-way ANOVAs with Bonferroni’s multiple comparisons tests (Microsoft Excel and GraphPad Prism), with statistical significance determined as being *p* < 0.05.

## Additional Information

**How to cite this article**: Foot, N. J. *et al*. Ndfip2 is a potential regulator of the iron transporter DMT1 in the liver. *Sci. Rep*. **6**, 24045; doi: 10.1038/srep24045 (2016).

## Supplementary Material

Supplementary Information

## Figures and Tables

**Table 1:** Hematological parameters in male and female mice fed a low or normal iron diet.

	Male				Female			
	Low iron diet		Normal diet		Low iron diet		Normal diet	
	Wild type	*Ndfip2*^*−/−*^	Wild type	*Ndfip2*^*−/−*^	Wild type	*Ndfip2*^*−/−*^	Wild type	*Ndfip2*^*−/−*^
Hemoglobin (g/L)	104.4 ± 11.5	111.0 ± 10.6	122.0 ± 6.7	125.4 ± 9.3	102.3 ± 1.5	113.2 ± 11.8	126.0 ± 8.1*	128.1 ± 5.8*
RBC count (x10^12^/L)	8.7 ± 0.3	8.9 ± 0.5	7.5 ± 0.3*	7.7 ± 0.7*	8.6 ± 0.3	8.9 ± 0.3	7.9 ± 0.5*	8.1 ± 0.4*
Hematocrit (%)	35.0 ± 3.0	37.0 ± 4.0	39.0 ± 1.0	40.0 ± 4.0	34.7 ± 1.2	38.0 ± 4.1	41.2 ± 1.7*	41.1 ± 1.5*
MCV (fl)	41.2 ± 2.4	41.3 ± 2.6	52.2 ± 1.0*	52.2 ± 0.9*	40.5 ± 2.2	42.8 ± 4.2	52.3 ± 1.1*	51.1 ± 0.9*
MCH (pg)	12.3 ± 0.7	12.4 ± 0.7	16.2 ± 0.4*	16.3 ± 0.9*	11.9 ± 0.5	12.8 ± 1.2	16.0 ± 0.1*	15.9 ± 0.2*
MCHC (g/L)	299.4 ± 9.5	302.0 ± 6.2	310.8 ± 10.9	313.0 ± 19.9	294.7 ± 3.5	298.8 ± 7.1	306.8 ± 7.4*	310.9 ± 5.6
RDW (%)	22.5 ± 2.7	21.9 ± 1.6	16.4 ± 1.1*	16.3 ± 1.0*	22.3 ± 1.0	22.1 ± 1.7	15.9 ± 0.2*	15.9 ± 1.2*

There are no significant differences in any of these parameters between wild type and knockout mice, or between males and females on any diet. *represents significant differences between low iron and normal diets within genders and genotypes. Data expressed as mean ± s.d.; n = 3–8. RBC; red blood cell; MCV, mean corpuscular volume; MCH, mean cell hemoglobin; MCHC, mean cell hemoglobin concentration; RDW, red cell distribution width.
