# Supplementary material for: Ndfip2 is a potential regulator of the iron transporter DMT1 in the liver
Source: Sci Rep. 2016 Apr 6;6:24045. doi: 10.1038/srep24045 (PMC4822147; doi:10.1038/srep24045)
Supplement: Supplementary Information [file srep24045-s1.pdf]

Ndfip2 is a potential regulator of the iron transporter DMT1 in the liver

Natalie J. Foot, Kelly M. Gembus, Kimberly Mackenzie and Sharad Kumar*

Centre for Cancer Biology, University of South Australia, c/o SA Pathology, PO Box 14, Rundle Mall, Adelaide, SA 5001, Australia

* Corresponding author: sharad.kumar@unisa.edu.au

Supplementary Material

Supplementary Table 1. Primers used for genotyping and qPCR. All primers were designed to amplify the mouse sequences.

Gene	Primer sequence (5'-3')
Ndfip2 genotyping primer "a"	CAGCATTGATGCATGGTCAGC
Ndfip2 genotyping primer "b"	GATTACAAATGCTCCTGCAGG
Ndfip2 genotyping primer "c"	CAACGGGTTCTTCTGTTAGTCC
TBP	F: CAAACCCAGAATTGTTCTCCTT
	R: ATGTGGTCTTCCTGAATCCCT
Fpn	F: TAAAGTGGCCCAGACGTCAC
	R: AGCAGACAGTAAGGACCCATC
HAMP	F: ACATTGCGATACCAATGCAGAA
	R: GCAACAGATACCACACTGGGA
